# The difficulties of measuring and improving physical activity in COPD

**DOI:** 10.1038/npjpcrm.2014.14

**Published:** 2014-05-20

**Authors:** Bernard Aguilaniu, Nicolas Roche

**Affiliations:** 1 Faculté de Médecine de Grenoble—Université Joseph Fourrier, Grenoble, France; 2 Department of Respiratory and Intensive Care Medicine, Groupe Hospitalier Cochin, AP-HP and Université Paris Descartes (EA2511), Paris, France

There is an article associated with this Editorial: 10.1038/npjpcrm.2014.3.

In the linked paper, Troosters *et al.*^1^ report the results of a randomised placebo-controlled trial assessing the effect of the long-acting anti-muscarinic agent tiotropium on lung function, physical activity (PA), health status and productivity in chronic obstructive pulmonary disease (COPD) patients with moderate lung function impairment (Global Initiative for Chronic Obstructive Lung Disease stage II) and no previous maintenance treatment. One noticeable result is that, despite clinically significant improvements in lung function (e.g., a mean difference in area under the curve from 0 to 3 h (AUC_0–3h_) forced expiratory volume in 1 s (FEV_1_) of 230 ml at the end of the treatment period), and statistically significant benefits in terms of health status and productivity, they observed no significant improvement in PA. Although some numerical differences were found, suggesting that lack of power could be involved, these findings question the significance and importance of PA as an evaluation tool and, more specifically, as a measure of treatment outcome in COPD.

Major determinants of quality of life impairment in COPD patients include handicap in daily life, and exacerbations. Handicap in daily life is the result of limitations in exercise capacity and/or tolerance, mostly as a consequence of ventilatory impairment (increased respiratory workload and decreased efficiency of inspiratory muscles), gas exchange abnormalities, and quantitative and qualitative peripheral muscle dysfunction (loss of muscle mass and enzymatic capacities).^2^ Other general (i.e. non respiratory) features can intervene, such as psychological status (see Figure 1).^3^ Handicap naturally leads to reductions in activities, including participation in social interactions. Indeed, several studies found that PA decreases in patients with COPD, being altered even in some patients with mild to moderate disease.^4^ PA is also related to prognosis: long-term all-cause and respiratory mortality and risk of hospitalisation and re-hospitalisation are notably increased in patients with lower levels of PA.^5^ In addition, some epidemiological studies have suggested that PA could even be a risk factor for the occurrence of COPD, which could be mediated by the systemic inflammation associated with a sedentary lifestyle. Altogether, physical inactivity is at least as detrimental in COPD patients than in the general population, where it was suggested to account for 6–10% of major non-communicable diseases.^6^

It thus seems logical to believe that restoring part of one’s physical capacities through improvements in ventilatory mechanics (with bronchodilators) and peripheral muscle function (with exercise training as part of rehabilitation programmes) should allow patients to have a more gratifying social life, which should then translate into measurable increases in PA. Several therapeutic trials have assessed the effect of these therapeutic approaches on PA. The vast majority tested exercise training. Altogether, results were inconsistent, especially in the long term. Accordingly, a recent systematic review by the PROactive consortium concluded that the evidence regarding deleterious consequences of low PA was convincing, while the level of evidence regarding determinants of PA including treatment effects was low.^7^ This low level of evidence was not related to a lack of studies but to methodological limitations such as cross-sectional design (preventing from determining the direction of observed relations) or lack of adjustment for potential confounders. As mentioned by the authors, although of low-level, evidence regarding associations between PA and hyperinflation, gas exchange, exercise capacity, dyspnoea, systemic inflammation, previous exacerbations, quality of life and self-efficacy was consistent, which was not true for associations with FEV_1_, forced vital capacity, body mass index, comorbidities and emotional status. Similarly, evidence from therapeutic studies was inconsistent. Of note, inconsistency was found not only for long-term effects but also for PA at the end of the rehabilitation programme.

There might be several explanations for the lack of robust effect of treatments on PA in patients with COPD. One hypothesis could be that methods of measurement are not sensitive enough. However, sensitivity might not be the issue, and/or measurement tools might also not be adapted to activities performed by COPD patients. Over the past 10 years, various tools have been developed to measure daily activities (mainly walking), and these are becoming an essential domain of the diagnosis of disability. Many subjective (questionnaires and diaries) and objective tools (indirect calorimetry, such as the doubly labelled water method) are regularly used for research purposes.^8,9^ Additionally, advances in microelectronics now allow, for an affordable price, the use of activity monitors with pedometers or actimeters that detect movements related to walking.^10^ These monitors can measure during several days the number of steps (and thus the distance) walked and the time, together with the metabolic expenditure beneficial for health.^11,12^ To complete these observations, it has also become possible to record more variables such as time spent sitting, standing time without moving, vertical metres walked, movements without displacements or movements of the upper body (thorax, upper limbs). These physiological variables can then be translated into metabolic expenditure. For this purpose, unit conversions can be made, accepting approximations on the metabolic efficiency or consulting the Compendium of PA regularly updated by Strath *et al.*^13^ The considerable amount of data that can now be collected highlights, on the one hand, the variability in the number of steps walked by a given subject from one period of time to another and, on the other hand, the extreme differences in psychomotor behaviour between patients with quite similar respiratory functional impairment (airflow obstruction, lung hyperinflation, diffusing capacity): some remain very inactive whilst others are in the upper ranks of recommendations for daily walking.

These observations illustrate that being engaged in a displacement activity (e.g., walking on flat ground) is influenced by many factors other than the respiratory functional disability. Clearly, exercise capacity/tolerance are far from being the sole determinants of PA, and their influence can be overwhelmed by factors such as way of life/habits or what could be called ‘behavioural/social deconditioning’. In a recent French survey in the general population, the two main factors limiting PA were lack of motivation (which was more pronounced in the more sedentary subjects) and lack of time.^14^ This might explain why correlations between PA and exercise testing using tests such as shuttle walking or 6-min walking are of rather low magnitude even when statistically significant. It might also explain why effects of rehabilitation on PA are not consistent even when PA is measured just after the exercise training programme, while lack of effects on the longer term could be due to tailing off.

The diversity of factors involved in determining disability is conceptually included in the definition of this term by the WHO committee in 2001 and was well described in a recent review,^15^ illustrating the limits of a purely numerical approach to PA, even if it is very accurate. Indeed, one can question the will to constantly increase measurement accuracy; in other words, according to the purpose of measurements, it is now time to consider how measurement data can be used to manage the excessive sedentary lifestyle of COPD patients, allowing one to define which type of information is needed, rather than thinking the other way around (i.e., I have to get the most precise information, I will see afterwards what I can do with it).

The linked study by Troosters *et al.*^1^ addressed this question by using several complementary approaches to assess PA in patients with moderate COPD. Although tiotropium did not statistically significantly increase the number of daily steps, the ‘global assessment of health’ by the patient and the physician were statistically improved after a few weeks of tiotropium treatment. This suggests that, in patients with respiratory deficiency, PA (as assessed by walking) is only one aspect of health status, which is highly dependent on various individual factors in a multifaceted psycho-social and geographical environment. Thus, relieving respiratory functional impairment may not be enough to increase daily PA, although at the beginning of disease progression, the deterioration of lung function may have been one of the main determinants of the reduction of walking activities, at least in some patients. To increase PA, practitioners may need to focus more on personal factors like the sensory and affective dimensions of dyspnoea, which may be facilitated by adding rehabilitation to the prescription of inhaled bronchodilators. Recognising the major impact of psycho-social determinants, health-care providers should also aim at initiating a new desire to diversify daily activities and perform more ‘moving activities’. This could allow a significant increase in movement-related energy expenditure. Considering the philosophy of desire’s construction implies a singular requirement for all tools measuring daily PA: they should be used first by the patient to self-manage his or her personal desire to move and walk daily. Furthermore, this singular requirement also raises the question of the real interest of the multiple measurements proposed nowadays to patients. In other words, tools measuring PA should be used for their potential to influence behaviour by allowing the patient to refine his own observations, and not only for their potential to change medical practice towards a more sophisticated science of human observation. The introduction of various sensors of PA into the lives of ordinary citizens is now a major target of many ‘well-being’ programmes. These strategies could probably be used in patients with mild to moderate COPD who are not heavily limited by ventilatory constraints during daily activities. For patients more severely limited by dyspnoea, but also by other functional impairments due to frequent co-morbidities (muscular atrophy, depression, cardio-vascular diseases, etc.), the influence of feedback from PA measures certainly needs to be evaluated in depth. This actually seems to be one of the objectives of the PROactive programme, which intends to evaluate daily PA through diversified tools without neglecting the role of their use by the patient himself.

In terms of therapeutic interventions, the inconsistency of long-term effects of rehabilitation on activity and health status led several clinicians and investigators to consider the need for more adapted programmes targeting behaviours as much as exercise performance. Disappointingly, a lifestyle activity intervention did not prove to be superior to traditional exercise training in COPD patients.^16^ Similarly, long-term supervised exercise programmes following rehabilitation do not seem to improve quality of life (or PA, although specifically measured in only one of the analysed studies) as compared to rehabilitation alone.^17^ These results should not lead to pessimism, since the use of more appealing activities such as urban walking circuits designed to offer cultural attractions and access to commercial areas could be of help.^18^ Patients’ associations may also play a role.^19^ Altogether, field programmes might be a useful complement to traditional exercise training.

Ultimately, in COPD patients, as in the general population, increasing PA likely requires much more than increasing physical capacities; both individually tailored and global public health actions are also necessary, as comprehensively outlined in recent reviews^20,21^ (see Figure 2).

## Figures and Tables

**Figure 1:**
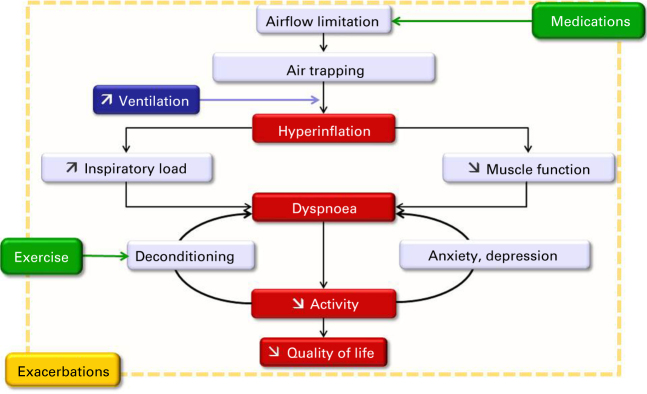
COPD-related mechanisms of quality of life and physical activity impairment.

**Figure 2:**
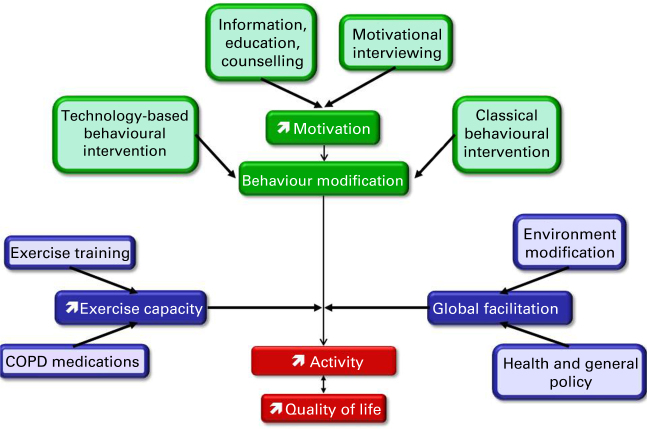
A putative model to improve physical activity levels in chronic obstructive pulmonary disease (COPD). Green zones contain triggers and blue zones contain facilitators.
